# Reconfigurable Magnetic Inhibitor for Domain Wall Logic and Neuronal Devices

**DOI:** 10.1021/acsnano.4c12503

**Published:** 2025-01-31

**Authors:** Christoph A. Durner, Andrea Migliorini, Jae-Chun Jeon, Stuart S. P. Parkin

**Affiliations:** 1 Center Nanoelectronic Technologies, Fraunhofer IPMS, An der Bartlake 5, Dresden 01109, Germany; 2 Max Planck Institute of Microstructure Physics, Weinberg 2, Halle (Saale) 06120, Germany

**Keywords:** racetrack, spintronics, domain wall motion, memory, logic, leaky integrate-and-fire

## Abstract

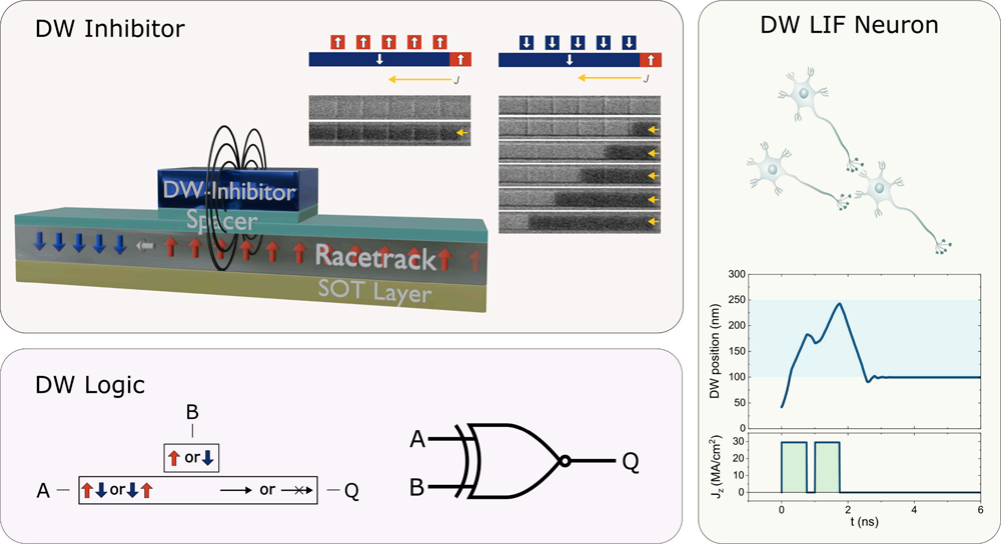

Spintronic devices based on the electrical manipulation of magnetic chiral domain walls (DWs) within magnetic nanowires promise advanced memory and logic with high speed and density. However, error-free positioning of the DWs along the magnetic nanowires is challenging. Here, we demonstrate reconfigurable domain wall logic and neuronal devices based on the interaction between the DWs and local magnetic inhibitors that are placed in the proximity of the magnetic nanowire. First, we investigate the effect of localized stray fields generated by a nanoscopic magnetic inhibitor on the motion of domain walls moved by current passing through the nanowires. We then show that the localized stray field is sufficient to inhibit or promote the current-induced propagation of chiral DWs depending on the state of the inhibitor. Further, we demonstrate that this allows for a DW-based logic XNOR gate and DW-based neuromorphic devices with leaky integrate-and-fire neuronal functions.

## Introduction

Chiral domain walls (DWs) in ferromagnetic (FM) systems are key elements for the implementation of advanced spintronic devices. In particular, the possibility of controlling chiral DWs in magnetic nanowires by electrically generated spin currents^1,2^ has led to the development of racetrack memory and logic technologies,^3−6^ which promise nonvolatile, energy efficient, and high-density devices with advanced functionalities.^7−13^ This technology is based on heavy metal (HM)/FM multilayered films with perpendicular magnetic anisotropy (PMA), which sustain efficient current-induced domain wall motion (CIDWM)^14,15^ resulting from the combination of an interfacial Dzyaloshinskii–Moriya interaction (DMI)^16,17^ that stabilizes Néel-type DWs^18−20^ and a spin–orbit torque (SOT) arising from spin currents generated in the heavy metal layer.^2,21^

The practical implementation of DW-based memory and logic devices requires precise positioning and local control of multiple DWs within the racetrack. For the precise positioning of DWs, embedding local pinning centers in a variety of ways has been considered, either by creating physical perturbations, such as geometrical notches^5,22^ or deformations,^23,24^ or by locally altering the device properties, such as magnetic anisotropy tuning,^25^ current density control,^11^ or modulation in the DMI or SOT.^26^ Some of these approaches have also been proposed or employed to implement DW filters and diodes,^27,28^ as well as complex logic gates based on DW propagation.^6,29^ However, the active reconfiguration of these perturbation elements for richer computational functionalities remains elusive. A promising approach for introducing reconfigurable perturbations in the system without undermining the device functionality is to exploit stray fields from magnetic elements located in the proximity of the racetrack device.^30−33^

Here, we present a reconfigurable magnetic inhibitor integrated in racetrack devices that allows for the realization of advanced functionalities based on the local manipulation of DWs within the racetrack. First, we investigate the interaction between the DWs and the stray field from a second FM layer located a few nanometers above the racetrack and how this affects the current-induced DW motion. We then fabricate devices with nanosized magnetic inhibitors that generate a local stray field within the racetrack. Our findings show that DWs are strongly affected by the stray field of the local magnetic inhibitors as it either promotes or prevents the passage of DWs based on their configuration. In addition, we show that by switching the magnetization direction of the local inhibitors, we can selectively induce a directional asymmetry in the current-induced DW motion. This enables the implementation of actively reconfigurable DW filters and diodes capable of performing logic operations and allows for functionalities of leaky integrate-and-fire neurons, which is of topical importance for spiking neural network operation in DW-based neuromorphic computing applications.

## Results and Discussion

**Figure 1:**
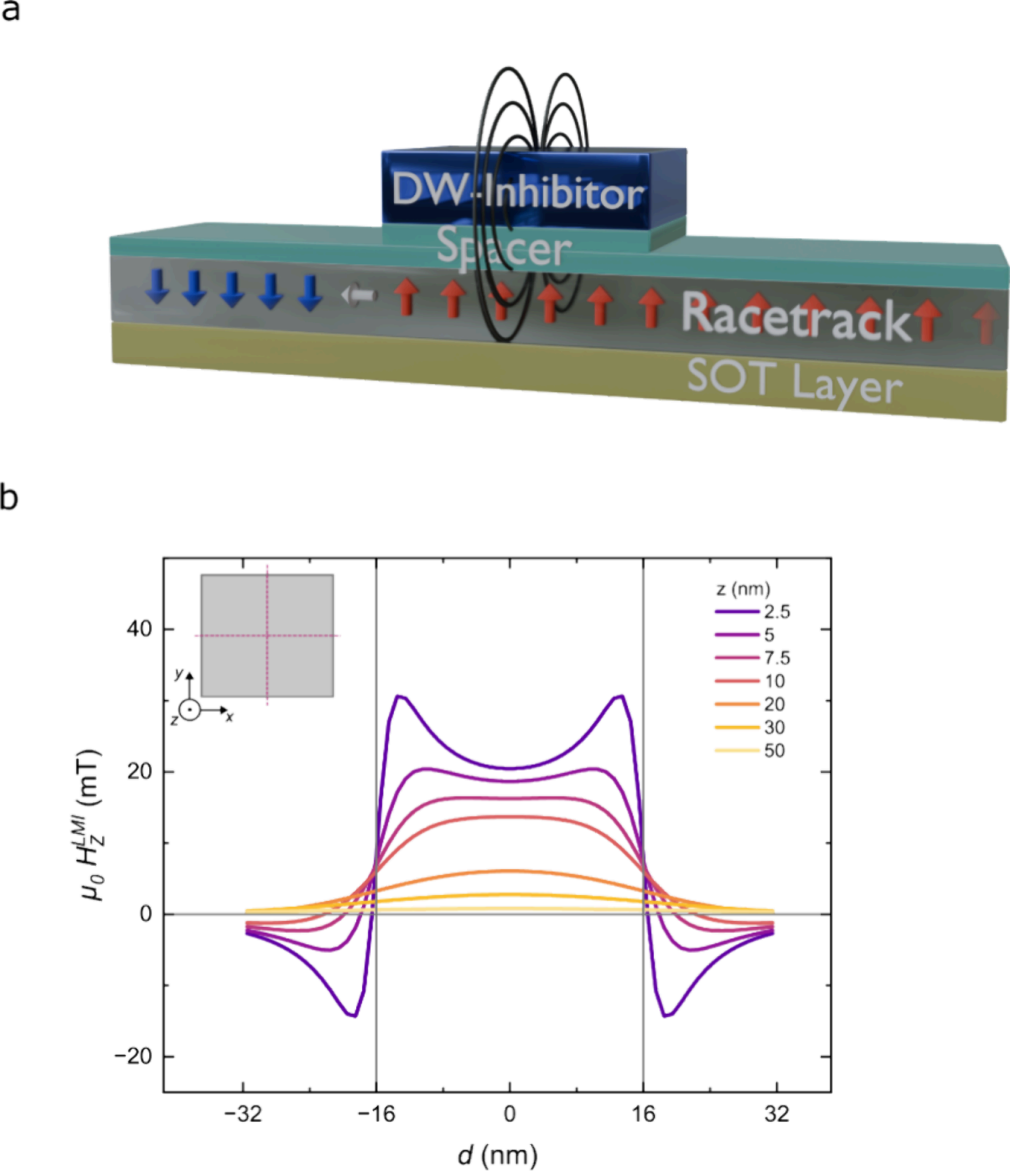
(a) Schematic illustration of the device, consisting of an SOT layer, a magnetic racetrack hosting the DWs, a spacer layer, and a nanostructured LMI. The racetrack contains ↓ (blue arrows) and ↑ (red arrows) magnetic domains separated by a ↓↑ Néel-type DW (white arrow). The stray field generated by the local magnetic inhibitor is represented by the black field lines. The magnetic inhibitor is magnetized either up or down. (b) Simulated *z*-component of the stray field, , generated by the local magnetic inhibitor as a function of the distance *d* from the inhibitor center along the *x* and *y* axes, for different spacer layer thicknesses *z*. The boundaries of the magnetic inhibitor are marked by gray vertical lines at *d* = −16 and 16 nm. The inset shows a top view schematic of the inhibitor. The red dashed lines indicate the position along which the stray field  is calculated.

A schematic illustration of the device concept is shown in Figure 1a. The racetrack nanowire consists of a thin FM layer with PMA, which hosts Néel-type chiral domain walls, and an HM layer, which is the source of the SOT required for efficient CIDWM. On top of the racetrack, a nanosized magnetic element with PMA, the local magnetic inhibitor (LMI), generates a stray field whose direction can be switched by changing the magnetization direction of the LMI. The racetrack and inhibitor are separated by a spacer layer, whose thickness is chosen to avoid direct magnetic coupling between the two. From micromagnetic simulations, we find that the stray field resulting from the LMI can significantly alter the energy landscape for DWs in the proximity of the inhibitor, thus enabling the local control of current-induced DW motion. The simulated profile of the *z*-component of the stray field, , along the length of the racetrack is plotted in Figure 1b, as a function of the spacer thickness (see Methods for details of the simulations). While the value of  decreases with spacer layer thickness, we find that quite strong stray fields are retained for thicknesses up to 10 nm, which confirms the technological feasibility of the local magnetic inhibitor concept.

**Figure 2:**
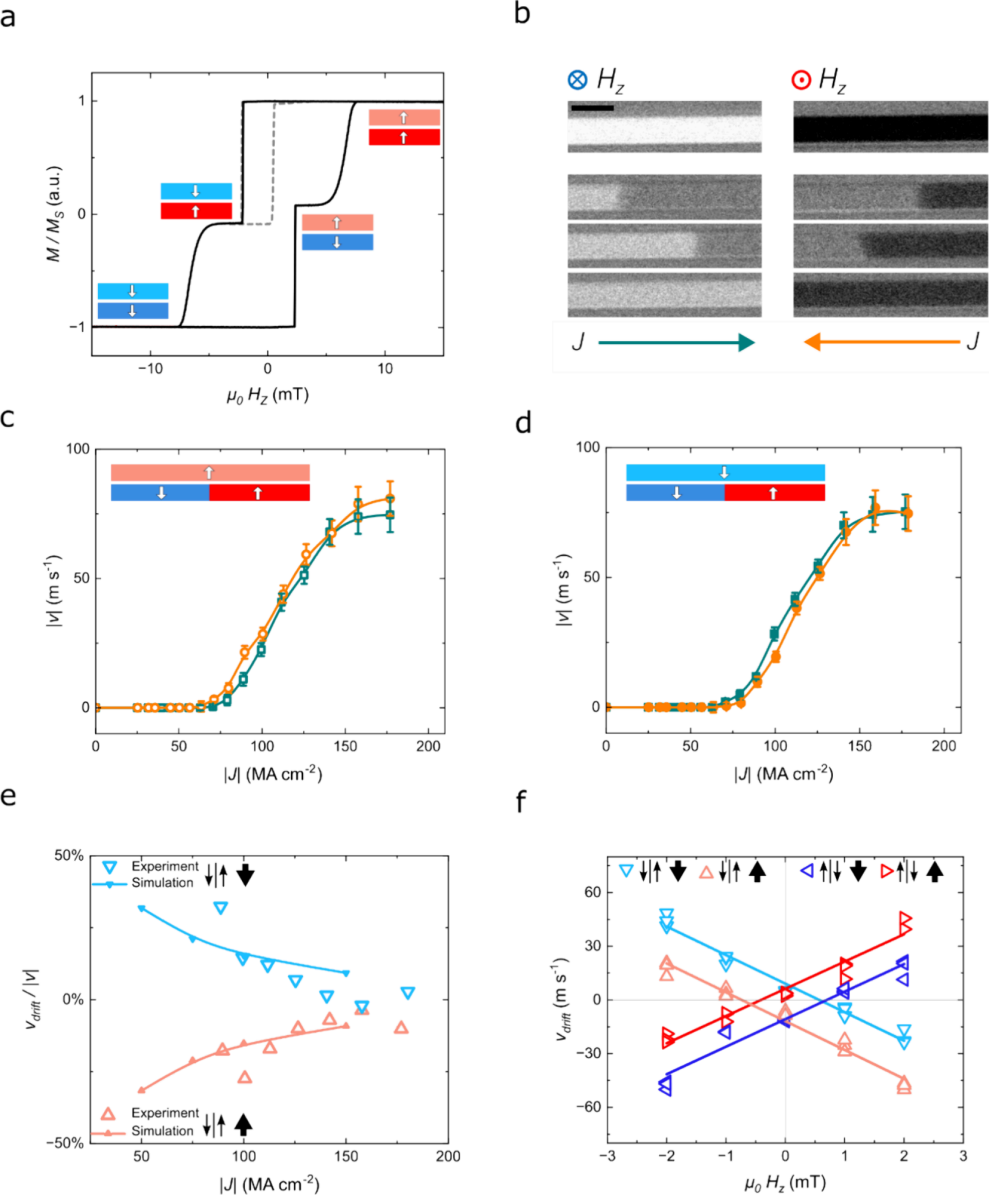
(a) Normalized out-of-plane magnetization loop of the racetrack plus inhibitor stack (solid black line) and minor loop for the inhibitor layer (dashed gray line) after postdeposition thermal annealing. The side-view schematics (shown as insets) depict the magnetization states of the inhibitor (top) and racetrack (bottom) magnetic layers. (b) Representative differential Kerr images of a 3 μm-wide device showing DW motion after current pulses are applied. The scale bar corresponds to 5 μm. Top panels: system saturated by external field along −*z* (↓) and +*z* directions (↑). Bottom panels: progressive shift of a ↓↑ DW upon applying positive (left) and negative (right) electrical currents. (c, d) Current-induced motion profiles of ↓↑ DW with the inhibitor layer being magnetized ↑ (c) or ↓ (d) for positive (green squares) and negative (orange circles) currents applied. (e) Normalized drift velocity vs current density for the inhibitor layer being magnetized ↑ (pink triangles pointing up) and ↓ (blue triangles pointing down) resulting from experiment (open symbols) and simulation (connected filled symbols). (f) Drift velocity vs applied magnetic field for ↓↑ (triangles pointing down or up) and ↑↓ (triangles pointing left or right) DWs and the inhibitor layer being magnetized ↑ (light and dark red triangles) and ↓ (light and dark blue triangles). Three data points are plotted for each experimental condition (field values and DW configuration).

To fabricate such a device, we deposited a multilayered thin film consisting of TaN(2)/[Pt(5)/Co(0.3)/Ni(0.7)/Co(0.3)]/[TaN(3)/Ta(2)]/[CoFeB(0.95)/MgO(2)]/TaN(3), where the thicknesses are given in nanometers. In the film stack, Pt/Co/Ni/Co, TaN/Ta, and CoFeB/MgO layers act as a racetrack, spacer, and magnetic inhibitor, respectively. To induce strong PMA in the CoFeB-based inhibitor layer, the film must undergo postdeposition thermal annealing (see Methods). Magnetometry measurements show that both magnetic layers have robust PMA, with square hysteresis loops, and that they switch independently with an external field, with the minor loop for the inhibitor layer being shifted by about 0.6 mT due to the magnetostatic interaction, confirming the magnetic decoupling of the two layers (Figure 2a). We then fabricated micrometer-sized devices to investigate CIDWM motion by Kerr microscopy (Figure 2b). The differential Kerr images show very bright (dark) contrast when both layers are saturated ↓ (↑) by an external magnetic field (top panels). Due to the large difference in coercivity, we can reconfigure each magnetic layer independently and nucleate a single DW in the lower racetrack layer (Figure S1). By sending electrical current pulses along the device, we can perform CIDWM without affecting the top inhibitor layer, as demonstrated by the dimer bright (dark) contrast of a ↓↑ DW moving to the right (left) in Figure 2b (bottom panels).

To investigate the influence of the stray field generated by the magnetic inhibitor on CIDWM, we extracted the DW velocity, *v*_DW_, for various current densities, *J*, for the two opposing saturation directions of the magnetic inhibitor layer (Figure 2c,d). By plotting *v*_DW_ vs *J*, the nonoverlapping data in Figure 2c,d indicate an asymmetry in the DW motion, which depends on the magnetization direction of the inhibitor layer. The stray field profile obtained by simulating a micrometer-sized inhibitor layer is rather uniform, and it is substantial enough (*H*_*z*_ ∼ 0.4 mT) to affect the domain wall motion,^15^ with nonuniformities of the stray field that only appear in proximity to the racetrack edges (Figure S2). Notice that, as one would expect, the DW motion is promoted in the direction that aligns the racetrack magnetization to the stray field from the magnetic inhibitor as this minimizes the magnetostatic energy of the system (Figure S3). To quantify the velocity difference at a given *J*, beyond the experimental error of the CIDWM measurements (Figure 2c,d), we repeatedly shifted a DW back and forth by current pulses with opposite polarity (see Methods). With repeated motion cycles, the DW progressively drifts in the direction promoted by the stray field (Figure S4). To quantify the drift, we define the drift velocity as follows:
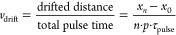
where *x*_0_, *x*_*n*_, *n*, *p*, and τ_pulse_ are the initial position, position after *n* cycles, number of cycles, number of pulses, and pulse length, respectively. Notably, the drift velocity induced by the stray field from the inhibitor layer is independent of the current density (Figure S5), analogous to the effect of an out-of-plane magnetic field.^15^ Note also that *v*_drift_ normalized to |*v*| is qualitatively in good agreement with micromagnetic simulations that reproduce this experiment (Figure 2e and Movie S1). From these simulations, we observe that the magnitude of *v*_drift_ depends also on the domain wall tilting induced by the DMI in our racetrack films^34^ (Figure S6). To further quantify the effect of the stray field from the magnetic inhibitor layer, we extract the drift velocity as a function of the magnetic field applied along the *z*-direction (Figure 2f). The DW motion is promoted in the direction that aligns the racetrack magnetization to the applied field, with a linear dependence between the drift velocity and the magnetic field strength. When the stray field is perfectly compensated by the applied external magnetic field, the drift velocity goes to zero. By linearly fitting the data, we can estimate a value of approximately 0.7 mT for the *z*-component of the stray field generated by the magnetic inhibitor layer.

**Figure 3:**
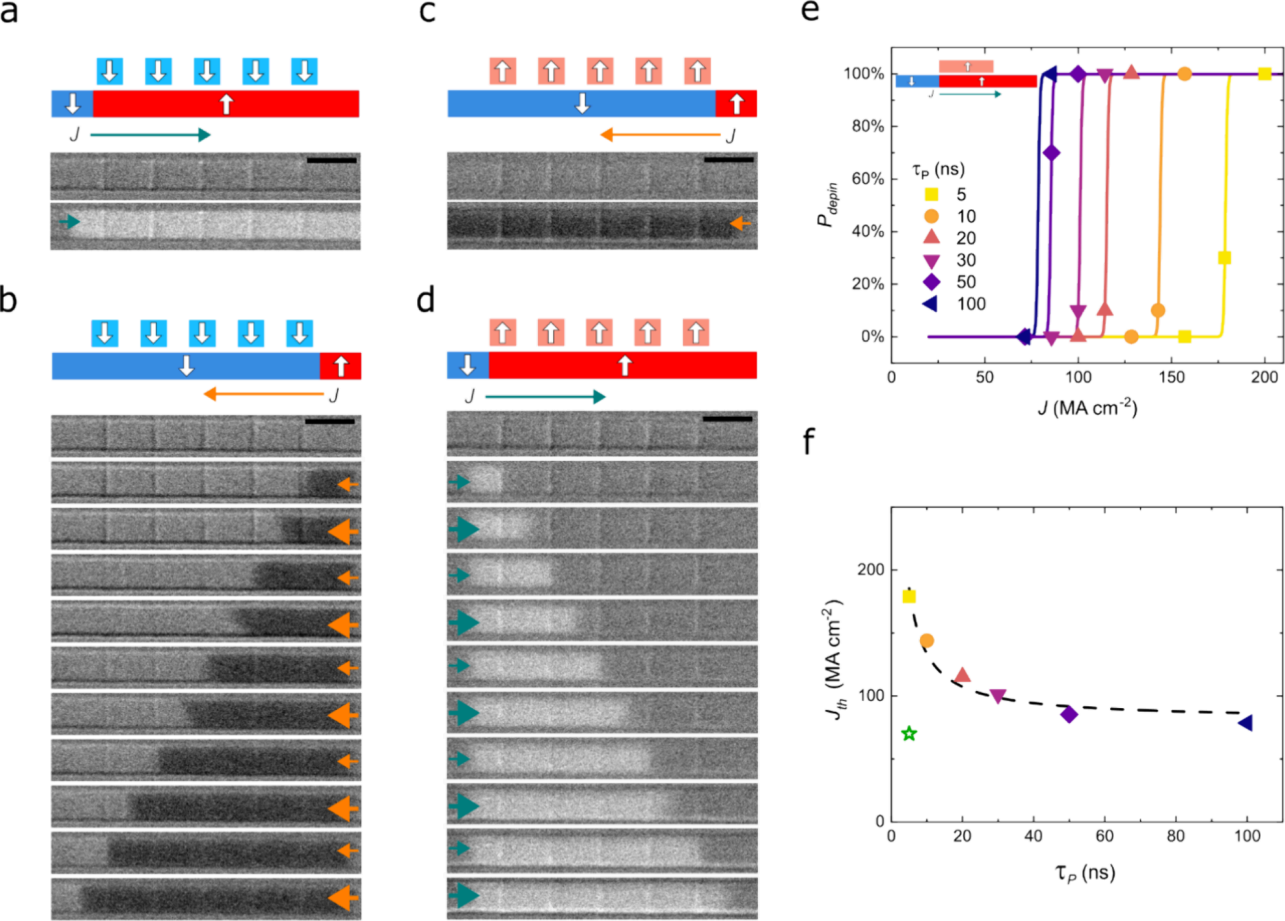
Side-view schematics and top-view differential Kerr images of a 3 μm-wide racetrack device with five 500 nm-wide inhibitors, all magnetized ↓ (a, b) or ↑ (c, d). The scale bar corresponds to 5 μm. A single ↓↑ DW is driven by positive (a, d) or negative (b, c) electrical current pulses, with low (small arrows) or high (large arrows) current density. For inactive inhibitors (a, c), low current density pulses are sufficient to drive the DW. For active inhibitors (b, d), we alternate low current density pulses to drive the DW in between inhibitors and high current density pulses to overcome the local pinning from the inhibitors. (e) Depinning probability as a function of current density and pulse length of a ↓↑ DW pinned at a 300 nm-wide inhibitor magnetized ↑, as represented in the inset side-view schematics. The lines represent the sigmoidal fitting to the data. (f) Threshold current density obtained from panel (e) as a function of the pulse length. The dotted line represents the hyperbolic fitting of the data. The green star corresponds to the current density needed to move the DWs in the absence of inhibitors.

We used conventional electron-beam lithography and ion-beam etching to shape the magnetic inhibitor layer and confine the resulting stray field, enabling precise and local manipulation of the DW motion. We fabricated a racetrack device, in which we patterned the inhibitor layer into a series of nanoscopic stripes that are perpendicular to the racetrack length and act as LMI for the DWs (Figure 3a–d). A key aspect of the device fabrication was to stop the etching process within the 5 nm spacer layer so that the ion beam does not damage the racetrack, which often causes the weakening of PMA. We confirm that the PMA of the racetrack layer is unaffected after the patterning process (Figure S7). We operate the device by displacing a single ↓↑ DW according to the four possible configurations shown in the schematics of Figure 3a–d, with all of the inhibitors saturated ↓ (a, b) and ↑ (c, d). When the DW is driven in the direction that aligns the racetrack magnetization with the stray field from the inhibitors, the DW can move freely along the racetrack without being affected by the presence of the inhibitors themselves. This is the case for the ↓↑ DW moving to the right underneath the ↓ DW inhibitors (a) and to the left underneath the ↑ DW inhibitors (c). On the contrary, when the DW is driven in the direction that opposes the racetrack magnetization to the stray field, the DW is pinned at each DW inhibitor along the track. To overcome the pinning potential, a higher current density is needed. This is the case for ↓↑ DW moving to the left underneath the ↓ DW inhibitors (b) and to the right underneath the ↑ DW inhibitors (d), in which we alternated low current pulses (∼120 MA/cm^2^) to drive the DW to the next inhibitor and higher current pulses (∼190 MA/cm^2^) to overcome the local pinning. Note that the same result with opposite polarity is obtained for ↑↓ DW (Figure S8).

These experiments clearly demonstrate that nanosized magnetic inhibitors act as high-precision DW pinning centers. To characterize the energy barrier imposed by the stray field from the magnetic inhibitors, we measured the DW depinning probability as a function of the current density and pulse length (Figure 3e). To account for the stochastic nature of the depinning process,^35^ we repeated every attempt 10 times by sending a single pulse with fixed current density and pulse length. Then, the depinning event was evaluated before the system was reset to the same initial condition. The threshold current density, *J*_th_, defined as the current density corresponding to 50% depinning probability, decreases with the pulse length (Figure 3f) and can be fitted by *J*_th_ – *J*_th0_ ∝ 1/τ_pulse_. Note that the threshold current density for the local magnetic inhibitor (∼190 MA/cm^2^) is nearly three times higher than the current density (∼70 MA/cm^2^) needed to move the DWs in absence of the magnetic inhibitor. From the fitting, we found that the corresponding energy barrier induced by the local magnetic inhibitor is Δ = 4.25 × 10^–18^ J at room temperature (see Methods), which is more than an order of magnitude higher compared to systems without the magnetic inhibitor.^9,36^ Note that the energy barrier can be tuned by appropriately designing the size of the inhibitor and/or the spacer layer thickness, as they both affect the resulting stray field (Figure 1b and Figure S11). A very important aspect is that the local magnetic inhibitor can be reconfigured by switching its magnetization direction, which can be achieved by several methods, for instance, by local Oersted fields,^37^ by SOT in cross-array geometries,^38^ or by spin-transfer torque (STT) in the current perpendicular-to-the-plane configuration.^39^ In this regard, by selectively manipulating the magnetization of nanosized inhibitors, we were able to confine a DW between two adjacent inhibitors with opposite configurations (Figure S9).

**Figure 4:**
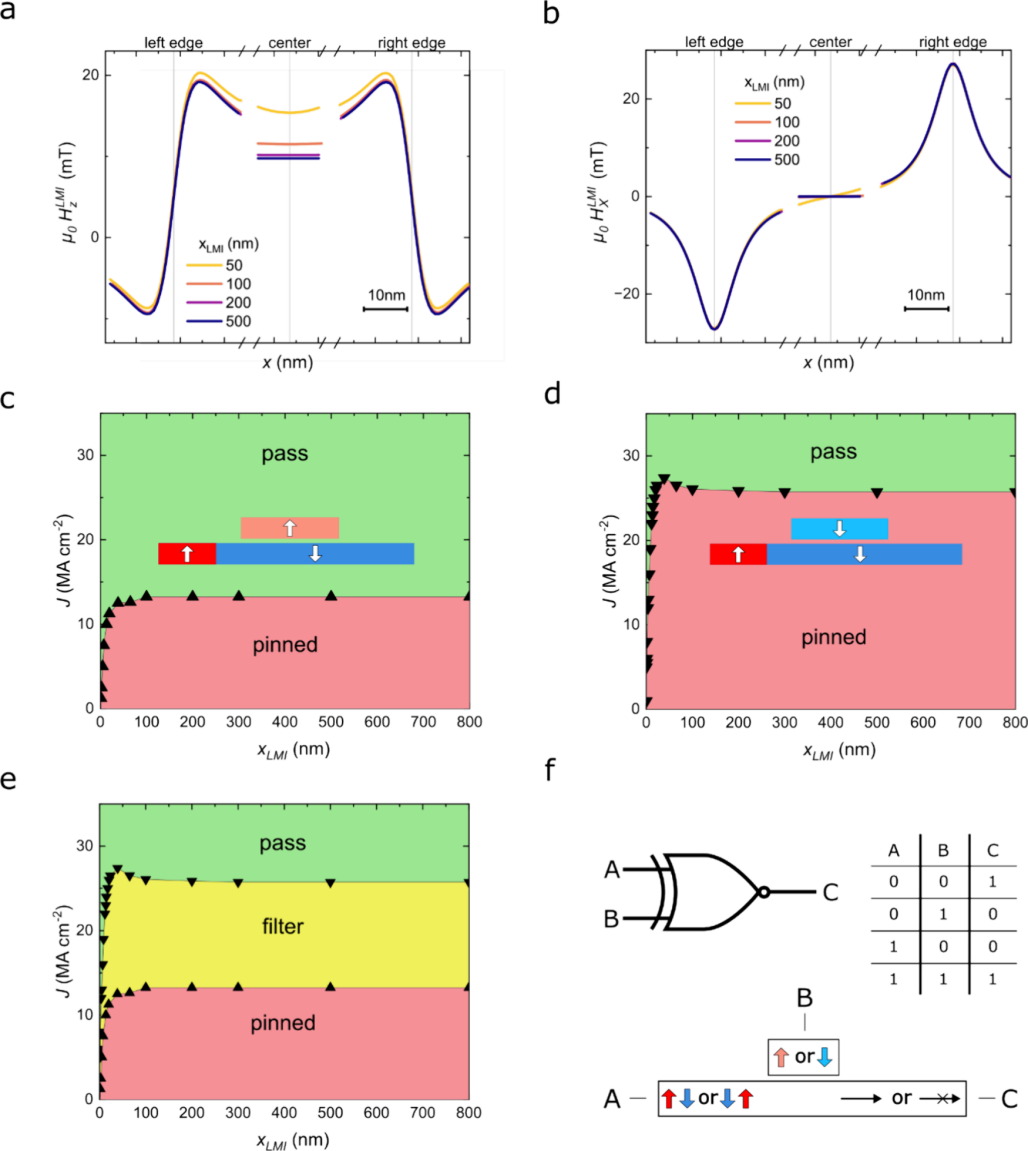
(a) *z*-Component and (b) *x*-component of the stray field generated by the local magnetic inhibitor, *H*^LMI^, along the racetrack length for different inhibitor lengths, *x*_LMI_. The three segments focus on the left edge, center, and right edge of the inhibitor. (c, d) Parameter space diagram illustrating the results of micromagnetic simulations for various current densities and inhibitor lengths, with the inhibitor magnetized ↑ (c) or ↓ (d). The green (red)-shaded area represents the parameter space for DW transmission (pinning). The insets show side-view schematics of the initial magnetic configuration of the device. (e) Combination of the diagrams from panels (c) and (d) where the yellow-shaded area represents the parameter space for DW filtering. (f) Representation and truth table of an XNOR gate based on a local magnetic inhibitor device.

To further unravel the influence of the magnetic inhibitor on current-induced DW motion in nanoscopic devices, we simulated the motion of a single DW as a function of the current density and dimensions of the local magnetic inhibitor (Figure 4; see Methods for details about simulations). First, we calculated the stray field along the racetrack length that results from magnetic inhibitors of different lengths, *x*_LMI_ (Figure 4a,b). The *z*-component of the stray field, , reaches its maximum values in close proximity to the edges of the magnetic inhibitor (a), making it either a favorable or unfavorable position for a DW, depending on its configuration, as confirmed by magnetostatic energy calculations (see Figure S10). Note that  at the center of the magnetic inhibitor increases as *x*_LMI_ decreases, leading to a higher energy barrier for the DW. On the other hand, the *x*-component of the stray field, , is independent of *x*_LMI_ (b) and reaches significant values in opposite directions at the two edges of the inhibitor. Note that high values of  can significantly affect the DW motion, as it would either increase or decrease the longitudinal torque given by the DMI in HM/FM bilayer racetracks.^15^ Note that the combination of the  and  peaks at the edge of the inhibitor could strongly contribute to the pinning of the DW. For the influence of the device width on the stray field from the inhibitor, see Figure S11.

Building upon these findings, we explore the device’s potential as a reconfigurable DW filter, controllable by external fields, local fields, or ideally, spin-polarized currents. Micromagnetic simulations were employed to investigate a wide range of inhibitor lengths and current densities, determining the conditions for domain wall transmission through the magnetic inhibitor, when magnetized ↑ or ↓ (Figure 4c,d). Notably, for application-relevant inhibitor lengths exceeding ∼30 nm, the threshold current density is nearly constant but remains strongly dependent on the magnetization state of the inhibitor (Figure S12). This characteristic enables the device to function as a DW filter within a broad range of current densities and inhibitor lengths (Figure 4e). These configurable DW filters open new avenues for realizing DW logic devices capable of logic operations such as the XNOR gate (Figure 4f). The filter itself functions as a programmable element that receives two inputs: A, encoded by the incoming domain wall type (↑↓ or ↓↑) and electrically displaced by CIDWM, and B, encoded by the local inhibitor magnetization (↑ or ↓), which can be electrically reconfigured by several means, as discussed in the previous section. The filter output reflects a logic “1” when domain wall transmission occurs. Conversely, the absence of transmission corresponds to a logic “0”. The electrical output is obtained by extracting the information about the eventual domain wall transmission, for example, by integrating nanosized anomalous Hall detectors^40^ or magnetic tunnel junctions.^41^

**Figure 5:**
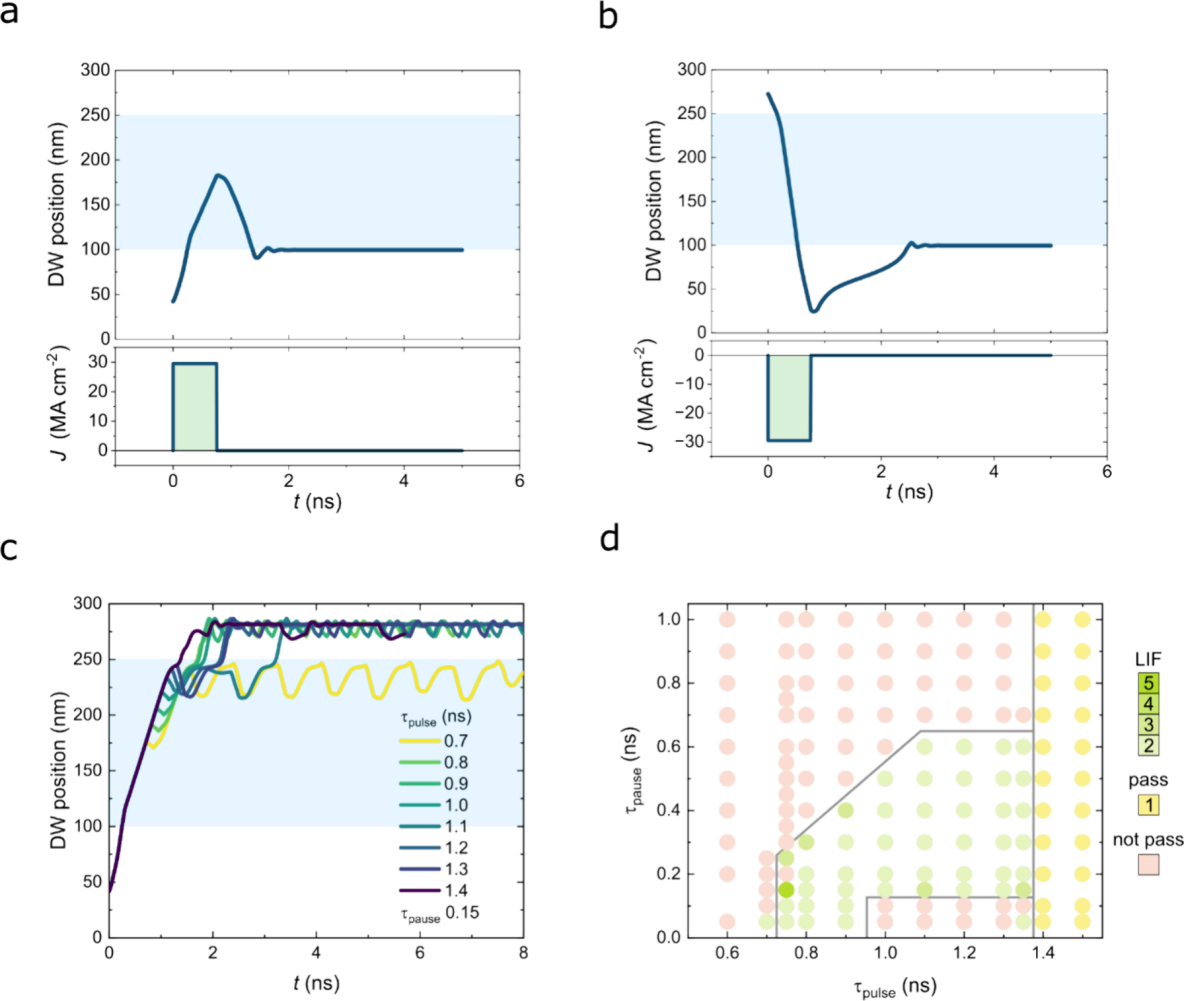
DW position and applied current pulse as a function of time, with the DW being injected underneath the magnetic inhibitor (light blue shade) in case of (a) DW repulsion and (b) DW acceleration configurations, respectively. Firing condition (pulse length and number), i.e., overcoming the magnetic inhibitor, of the DW neuron. (c) Pulse length (τ_pulse_)-dependent time-series of the DW position for a train of current pulses (τ_pause_ = 0.15 ns), showing that the DW overcomes the magnetic inhibitor with a response that emulates the leaky integrate-and-fire function of a neuron. (d) Parameter space diagram for various pause and pulse lengths for a given *J* = 29.5 MA/cm^2^. The colored symbols represent the number of pulses needed to overcome the magnetic inhibitor, i.e., threshold condition. Three regions are identified (separated by gray lines). The green symbols indicate the conditions for LIF functionality. The yellow symbols indicate the conditions for which the DW overcomes the inhibitor with a single pulse. The red symbols indicate the conditions for which the DW cannot overcome the inhibitor. Note that, when the DW is beyond the magnetic inhibitor, a current pulse with opposite polarity can easily reset the DW neuron (b).

Finally, we demonstrate and validate a leaky integrate-and-fire (LIF) neuron function, which is of importance for developing DW-based neuromorphic computing,^42−49^ in nanoscopic inhibitor-integrated racetrack devices using micromagnetic simulation. We find that, when the DW propagation distance is shorter than the length of the inhibitor, the DW can be repelled or accelerated depending on the relative magnetic configuration of the DW and the inhibitor (Figure 5a,b). In the case of DW repulsion from the inhibitor (Figure 5a), when the DW is stopped underneath the inhibitor with the application of a single current pulse (750 ps), the DW relaxes back to the edge of the magnetic inhibitor due to the interaction with its stray field. This self-reset process corresponds to short-term plasticity and leakiness over time in the absence of an additional external perturbation. On the contrary, in the case of the inhibitor configuration for DW acceleration (Figure 5b), even with a single short pulse, the DW passes the magnetic inhibitor due to the field-induced motion. For the DW repulsion configuration, when a sufficient number of current pulses is serially applied within a period shorter than the complete relaxation time, the DW can overcome the energy barrier provided by the inhibitor. If one considers the inhibitor as a threshold boundary, such a DW-overcoming process via successive current pulses can be considered to be equivalent to “integration and firing”. The pulse conditions for the DW neuron firing are shown in Figure 5c,d. Note that, in the case of τ_pause_ = 0.1 ns, effects such as domain wall tilting and inertia^50^ are responsible for the nonlinear behavior when τ_pulse_ is between 1.0 and 1.4 ns (see Movie 2). The pause time dependence with two pulses of a given pulse length is shown in Figure S13 for further information. When the DW overcomes the inhibitor, it can be self-reset by applying a depression pulse, as shown in Figure 5b.

## Conclusions

In conclusion, we have reported the local manipulation of the CIDWM using stray fields from nanosized magnetic inhibitors. We experimentally demonstrated that a second ferromagnetic layer located a few nanometers above the racetrack influences the DW motion according to its magnetization direction, emulating an external magnetic field. We then realized a novel DW device with nanosized inhibitors on top of a racetrack that exploits the stray field from the local inhibitors to precisely manipulate the DW motion. We demonstrated that such a device can function as a reconfigurable DW filter, which selectively permits the transmission of DWs based on their magnetization configuration. Building upon this ability to manipulate DWs, we then discussed the potential of the local magnetic inhibitor for an XNOR DW logic gate and neuronal devices. This work not only introduces advanced functionalities in DW-based logic and memory devices but also opens exciting avenues for neuromorphic computing through the LIF function in our devices.

## Methods

### Sample Preparation

For the film growth, we used a home-built sputtering system with a base pressure of <10^–9^ Torr. The films were deposited at room temperature on thermally oxidized Si(100) wafers by DC magnetron sputtering at an Ar pressure of 3 mTorr, with the exception of the TaN layers, which were grown by reactive sputtering in Ar/N_2_ environment, the MgO layer, which was grown by RF magnetron sputtering, and the Ta layer, which was grown by ion-beam deposition. The as-grown films were annealed in an argon environment at 300 °C for 20 min using a UniTemp Rapid Thermal Vacuum Process Oven (UniTemp, RTP-100).

### Magnetic Characterization

Vibrating sample magnetometry (VSM) was used to measure the magnetic properties of blanket films at room temperature by using a Lakeshore VSM 8600. The applied magnetic field ranged from −2 to +2 T.

### Device Fabrication

Nanowires with dimensions of 40 μm in length and 3 μm in width were fabricated using conventional maskless photolithography (MLA150, Heidelberg) and ion-beam etching (scia Coat 200, scia Systems) techniques. For the devices with a magnetic top layer (magnetic inhibitor layer), a single patterning step with negative photoresist (ARN 4340, Allresist) and etching was sufficient. For the devices with nanosized magnetic inhibitors, electron-beam lithography (JBX-8100FX, JEOL) with negative resist (ARN 7520-18, Allresist) was used to pattern the ferromagnetic islands. The subsequent etch step is stopped precisely in the TaN/Ta spacer layer, controlled by secondary ion mass spectrometry. VSM measurements confirmed a minimal effect of the etching process on the magnetic properties of the Co/Ni/Co layer. Finally, a second photolithography step with a negative resist and etching defined the final nanowire track.

### Kerr Microscopy and DW Motion Measurements

The characteristics of current-induced domain wall motion were measured using magneto-optical Kerr microscopy (customized system, evico magnetics) at room temperature. For the nanosecond-current pulses, a pulse generator (PSPL10300B, Tektronix) with a 300 ps rise time was used. A differential Kerr imaging technique enabled the measurement of the distance traveled by the DWs upon sending a series of nanosecond-long current pulses along the racetrack and the calculation of the DW velocity, *v*_DW_. The error bar for such a measurement is calculated as the ratio between the resolution limit of the Kerr microscope, which we estimate to be 1 μm, and the total pulse duration. To achieve a precise measurement of the drift velocity, a series of current pulses with opposite polarity was applied to ↓↑ DWs positioned in the track. These pulses caused the DW to shift back and forth repeatedly. The effective distance traveled by the DW was then measured. Combining this distance with the total pulse duration allowed for calculation of the drift velocity. This process was repeated three times at each current density and for each configuration to account for potential pinning effects. To ensure reliable determination of the energy barrier, depinning measurements were repeated 10 times with the initial DW precisely positioned directly in front of the DW inhibitor.

### Calculation of the Energy Barrier

From the depinning probability, the threshold current density *J*_th_ was extracted for varying pulse lengths. By plotting *J*_th_ as a function of pulse length τ_pulse_, a hyperbolic fit yields *J*_th_^0^ = 81.3 ± 5.4 MA/cm^2^. In the adiabatic STT model,^51,52^ the energy barrier is described by Δ = 2Ωλ*K*_d_, where Ω, λ, and *K*_d_ are the cross-sectional area, DW width parameter, and effective DW anisotropy, respectively.^36^ This energy barrier gives rise to a threshold current density to depin the DW from the pinning center—created by the magnetic inhibitor in our study. The threshold current density then can be calculated by , where *e*, γ, *p*, and μ_B_ are the electron charge, gyromagnetic ratio of the electron, spin polarization, and Bohr magneton, respectively. Therefore, the energy barrier can be calculated from the threshold current *I*_th_ by Δ = 2*p*μ_B_*I*_th_/*e*γ. For the calculations, a spin polarization of *p* = 0.5 was used.^15^

### Micromagnetic Simulations

Micromagnetic simulations were conducted using the GPU-based open-source software MuMax3.^53^ A uniform discretization cell size of 1 nm^3^ was employed. The simulated geometry varied in length along the *x*-direction from 300 to 1024 nm, with a constant width of 64 nm and a thickness of 4 nm. The following magnetic parameters were used in the simulations: For the track, the parameters used are a thickness of 1 nm, a saturation magnetization of 5.8 × 10^5^ A/m, an exchange stiffness of 1.5 × 10^–11^ J/m, an interfacial Dzyaloshinskii–Moriya strength of 3 × 10^–3^ J/m^2^, a perpendicular anisotropy constant of 8 × 10^5^ J/m^3^, and a Landau–Lifshitz damping constant of 0.1. Note that the DW filter functionality remains similar for *D* = 0.5 × 10^–3^ J/m^2^, allowing for a wide range of materials for the racetrack layer (Figure S14). The spacer thickness is 2 nm (vacuum). For the DW inhibitor, the parameters used are a thickness of 1 nm, a saturation magnetization of 8 × 10^5^ to 9.05 × 10^5^ A/m, an exchange stiffness of 1 × 10^–11^ J/m, a perpendicular anisotropy constant of 1 × 10^6^ J/m^3^, and a Landau–Lifshitz damping constant of 0.1. Note that, due to extrinsic pinning in the real device, the threshold current densities of the experiments are higher than in simulations.

## Supporting Information Available

Figure S1: optical image and P-MOKE hysteresis loop of a racetrack-inhibitor device; Figure S2: simulated stray field generated along the racetrack width; Figure S3: demagnetization energy calculated via micromagnetic simulations; Figure S4: differential Kerr microscopy images to investigate the drift velocity; Figure S5: drift velocity for various current densities; Figure S6: simulated domain wall tilting and drift velocity as a function of the interfacial DMI constant; Figure S7: VSM loops of the racetrack-inhibitor film before and after ion-beam etching; Figure S8: differential Kerr images of ↑↓ DW moved by positive or negative electrical current pulses; Figure S9: demonstration of the reconfigurability of DW inhibitors; Figure S10: energy landscape of a DW under the influence of a 100 nm DW inhibitor; Figure S11: stray field generated by the LMI along the racetrack length for different widths; Figure S12: threshold current density for a ↑↓ DW propagating below a magnetic inhibitor; Figure S13: Pause time-dependent leaky integrate-and-fire behavior with two consecutive pulses; Figure S14: micromagnetic simulation of DW filter operation for *D* = 0.5 mJ/m^2^ (PDF)
Movie S1: drift velocity experiment in micromagnetic simulations (MP4)
Movie S2: micromagnetic simulations of the LIF functionality (MP4)
