# Supplementary material for: Reconfigurable Magnetic Inhibitor for Domain Wall Logic and Neuronal Devices
Source: ACS Nano. 2025 Jan 31;19(5):5316–25. doi: 10.1021/acsnano.4c12503 (PMC11823606; doi:10.1021/acsnano.4c12503)
Supplement: Supplementary file 1 — nn4c12503_si_001.pdf [file nn4c12503_si_001.pdf]

Supplementary information

Reconfigurable Magnetic Inhibitor for Domain Wall Logic and Neuronal Devices

Christoph A. Durner[1,2,3], Andrea Migliorini[2,3], Jae-Chun Jeon[2*], and Stuart S. P. Parkin[2*]

[1]Fraunhofer IPMS, Center Nanoelectronic Technologies, An der Bartlake 5, 01109 Dresden, Germany

[2]Max Planck Institute of Microstructure Physics, Weinberg 2, 06120 Halle (Saale), Germany

[3]These authors contributed equally to this work

*Corresponding authors: jae-chun.jeon@mpi-halle.mpg.de, stuart.parkin@mpi-halle.mpg.de

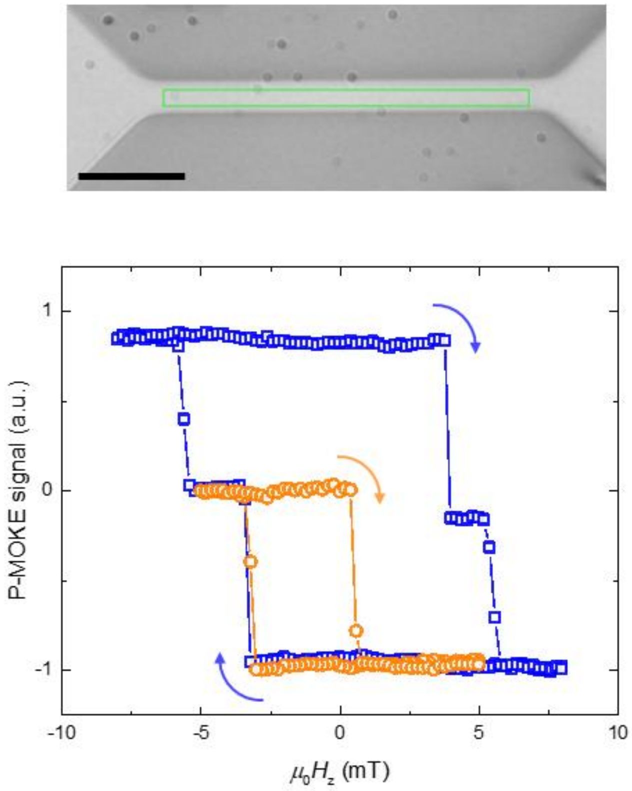

Figure S1. Optical image (top) and P-MOKE hysteresis loop (bottom) of a 3-µm-wide racetrack-inhibitor device. The P-MOKE signal is extracted from the area within the green rectangle. The arrows indicate the magnetization reversal of the inhibitor layer during the minor (orange) and full (blue) hysteresis loops. The minor loop of the inhibitor is shifted by about 1.3 mT due to the stray field generated from the racetrack. Scale bar corresponds to 10 µm.

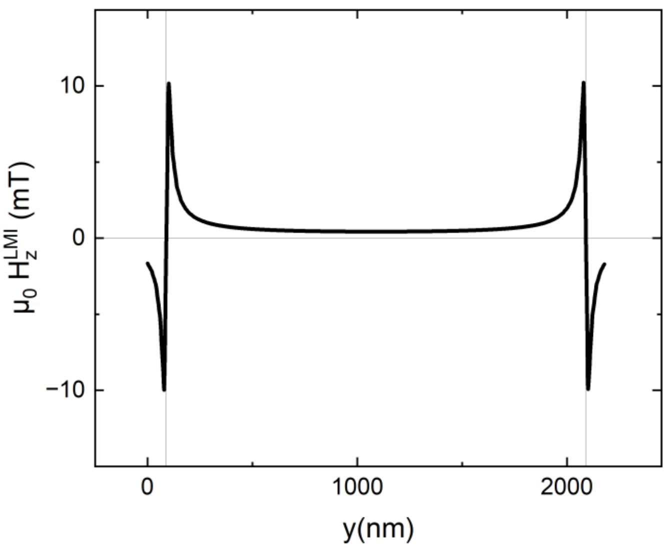

Figure S2. Simulated z-component of the stray field generated along the racetrack width *y*, by a local magnetic inhibitor of 4 µm length, 2 µm width and 1 nm height. The grey vertical lines represent the edges of the inhibitor.

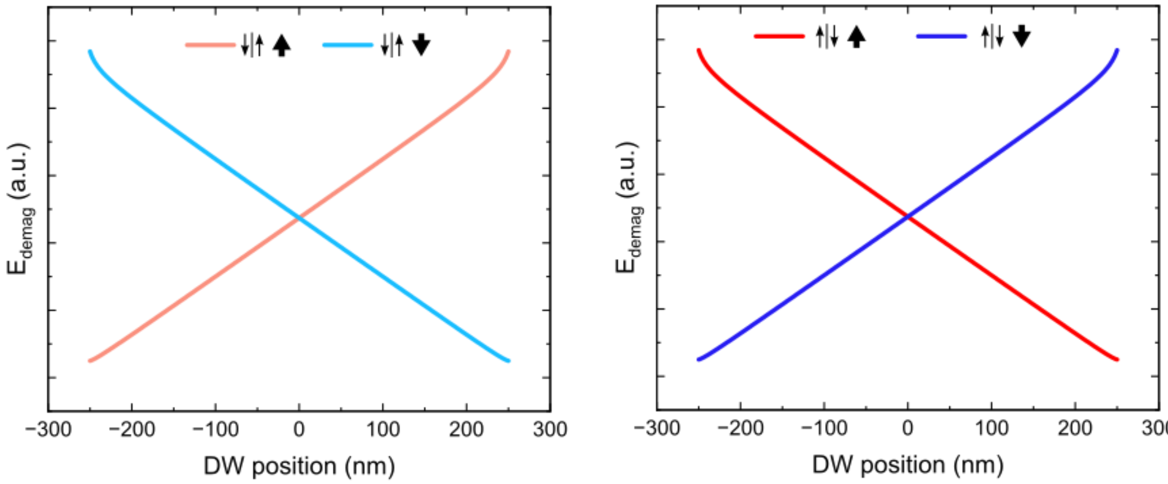

Figure S3. Demagnetization energy, E_{demag}, vs DW position calculated via micromagnetic simulations. The global minimum (maximum) for E_{demag} is reached when the magnetization direction of the racetrack and the magnetic inhibitor layers are parallel (antiparallel).

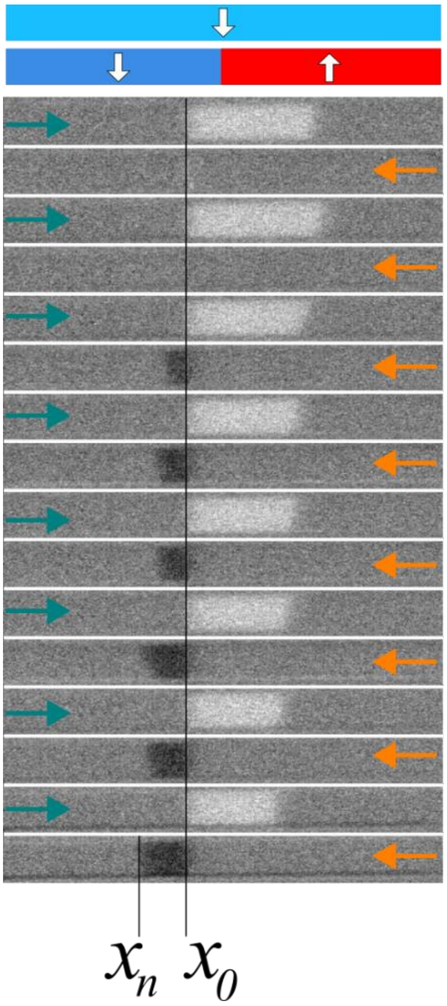

Figure S4. Differential Kerr microscopy images of a racetrack device under the influence of a magnetic inhibitor layer, according to the side-view schematics, upon cycling current pulses in opposite direction. Each Kerr image shows the distance travelled by the DW after current pulses were applied for 8 cycles. The DW is initially at position x_0 and is driven alternately by a number of positive current pulses p with a length τ_{pulse} to the right and by the same number of negative current pulses p with the same pulse length τ_{pulse} to the left. After n such operations, the DW has reached the position x_n. The DW drift velocity is then defined as $v_{drift} = \frac{x_n - x_0}{n \cdot p \cdot \tau_{pulse}}$.

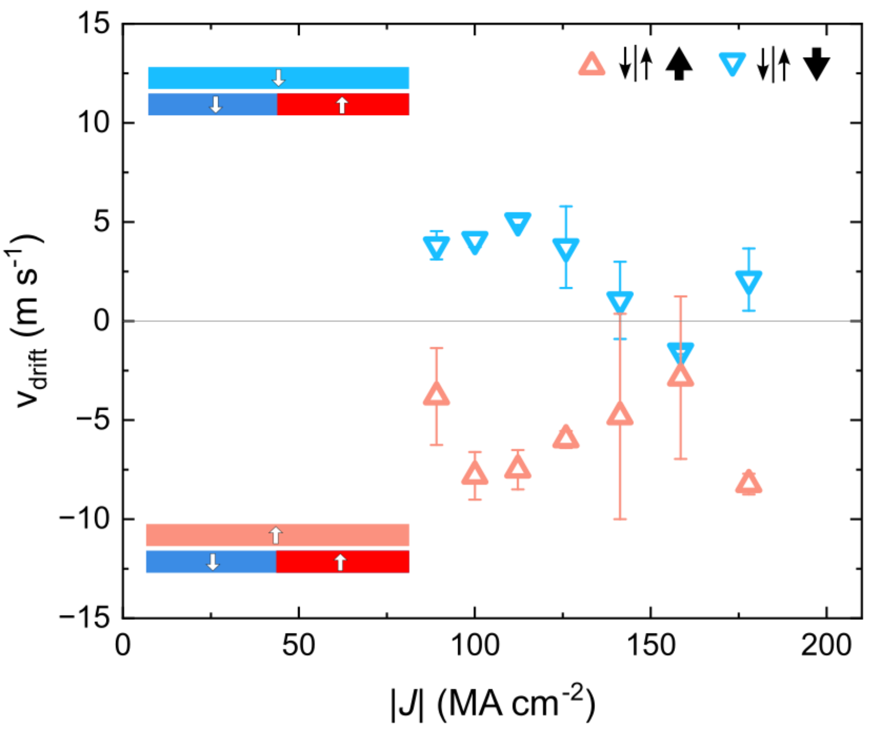

Figure S5. Drift velocity for various current densities for a ↓↑ DW with top layer magnetized down (blue open triangle pointing downwards) and up (red open triangle pointing upwards)

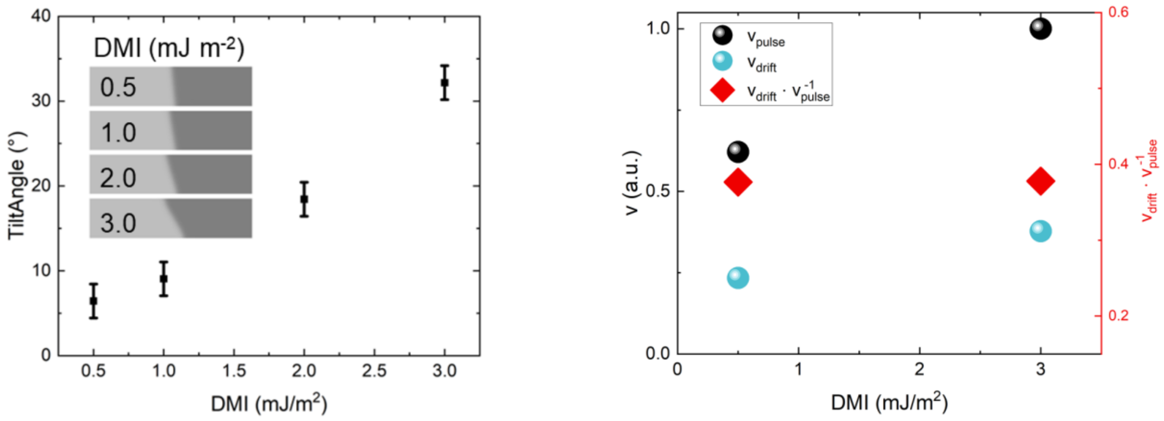

Figure S6. Simulated domain wall tilting (left) and drift velocity (right) as a function of the interfacial DMI constant with $|J|$ = 20 MA/cm^2.

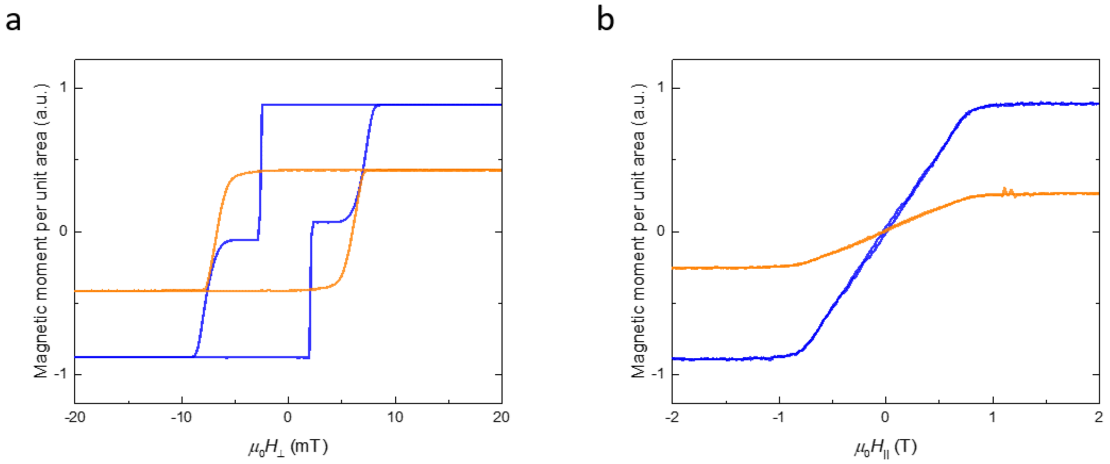

Figure S7. Out-of-plane (a) and in-plane (b) VSM loops of the racetrack-inhibitor film before (blue) and after (orange) ion-beam etching, confirming that the top inhibitor layer has been successfully removed without undermining the PMA of the racetrack layer.

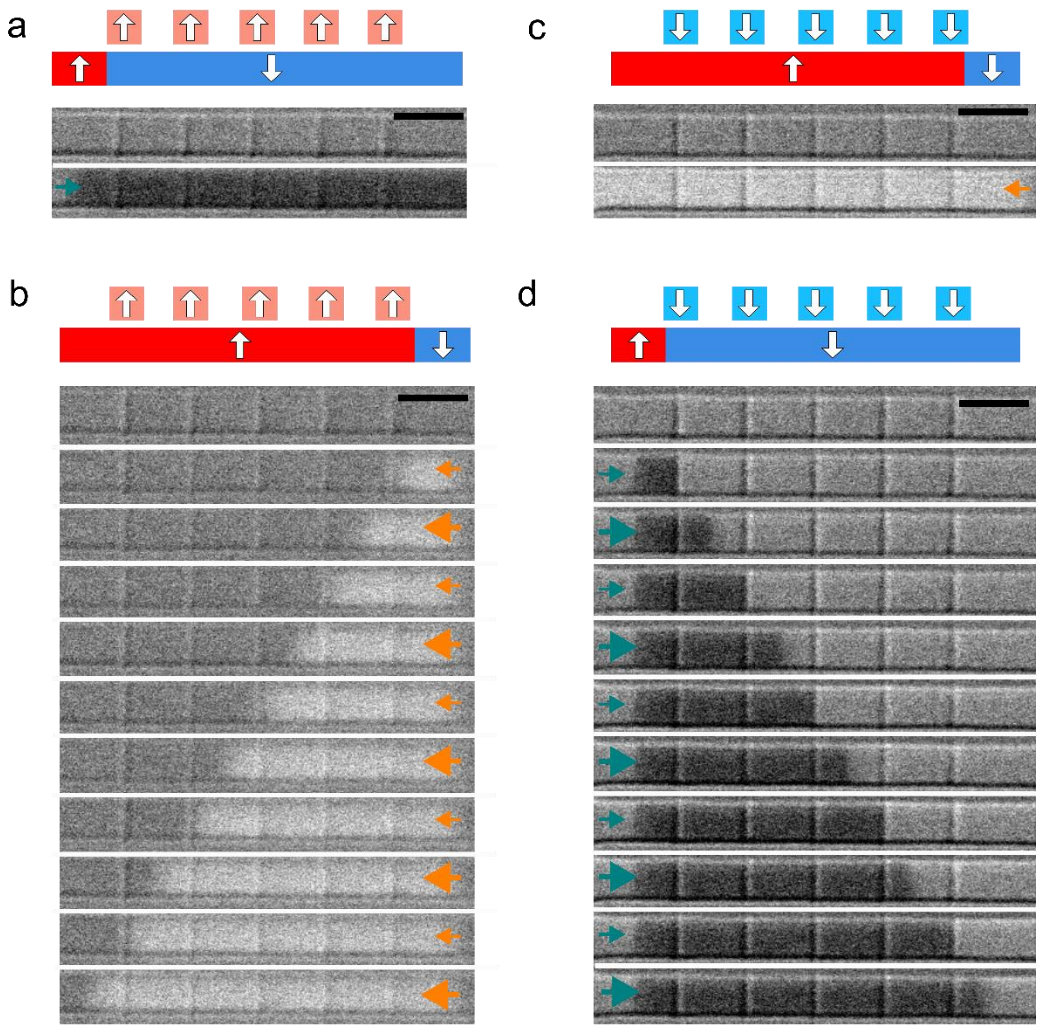

Figure S8. Differential Kerr images of ↑↓ DW moved by positive (a, d) or negative (b, c) electrical current pulses. The DW-inhibitors are 500 nm in length and magnetized up (a, b) or down (c, d). For (a) and (c), between the first and second Kerr image, 1000 pulses of low current density were applied. For (b) and (d), alternately, 1000 pulses of low current density and 10 pulses of high current density were applied. The schematics at the top of each panel provide a side view of the device with the magnetic configuration of the DW-inhibitors and the type of the DW. Scale bars correspond to 5 μm.

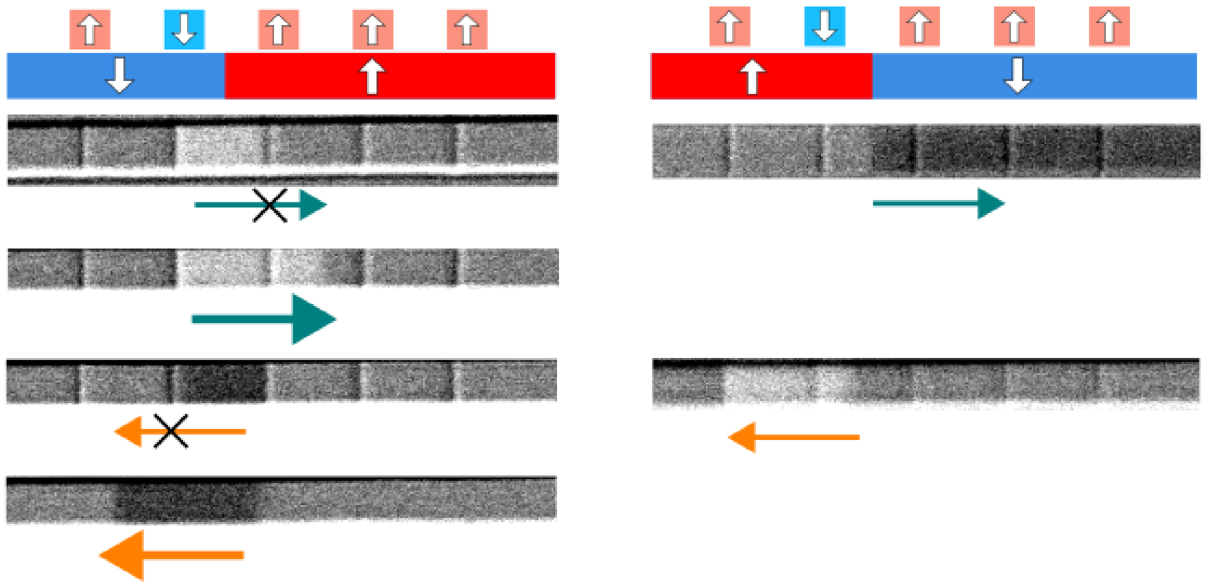

Figure S9. Demonstration of reconfigurability in an array of five 300 nm long DW inhibitors, for which the second one is magnetized down, while the others are magnetized up. A DW is located between 2nd and 3rd DW inhibitors. In the case of a ↓↑ DW (left panel) it cannot pass the 3rd inhibitor with low current densities to the right, nor the 2nd inhibitor with low current densities to the left. For higher current densities however, the DW can pass to the right and to the left. A ↑↓ DW (right panel) can pass to the right and to the left with low current densities. The device initialization was achieved by careful application of an out-of-plane magnetic field, exploiting the small variation in coercivities of the nanosized inhibitors, which results from slight variability in the fabrication. The desired configuration of the inhibitors was confirmed by Kerr contrast.

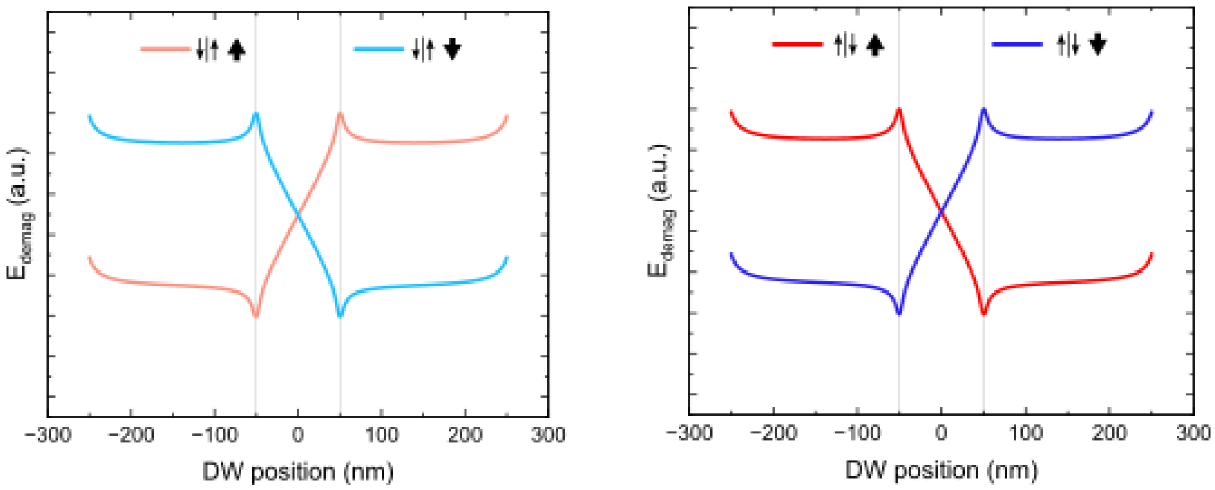

Figure S10. Energy landscape of a DW under the influence of a 100 nm DW inhibitor (the edges of the inhibitor are marked with grey lines), the minimum of the demagnetization energy is at the edge of the DW inhibitor, so that the up (down) domain is aligned with the up (down) magnetization of the DW inhibitor. This energy minima allows the precise positioning of the DW in nanoscopic racetrack devices. The maximum occurs at the edge of the DW inhibitor, if the domain under the inhibitor is oppositely magnetized to that of the DW inhibitor.

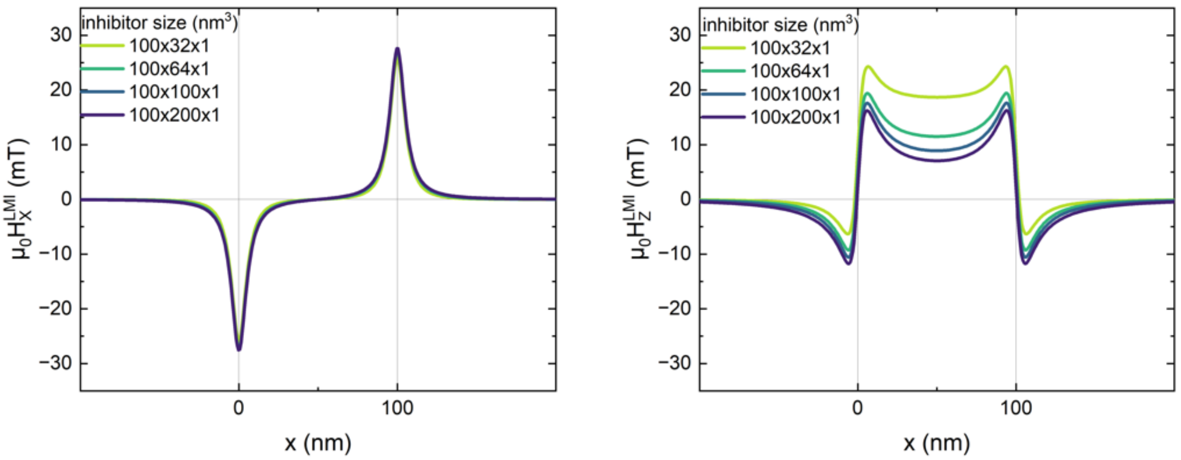

Figure S11. (Left) x- and (right) z-component of the stray field generated by the local magnetic inhibitor along the racetrack length for different racetrack-inhibitor widths, y_{LMI}. The x-component of the stray field is largely independent of the device width, while the z-component increases as the racetrack-inhibitor device gets narrower, similarly to H_z^{LMI} vs x_{LMI} (Fig. 4a).

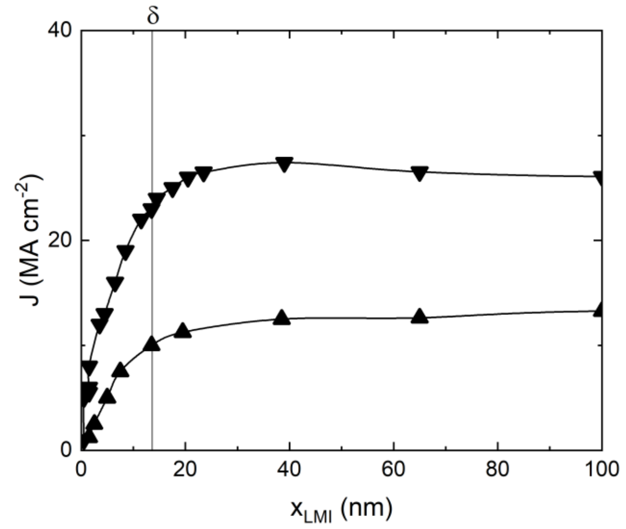

Figure S12. Threshold current density for a ↑↓ DW propagating below a magnetic inhibitor magnetized ↑ (up triangles) or ↓ (down triangles), as a function of the inhibitor length. The vertical line corresponds to the calculated domain wall width δ of 14 nm.

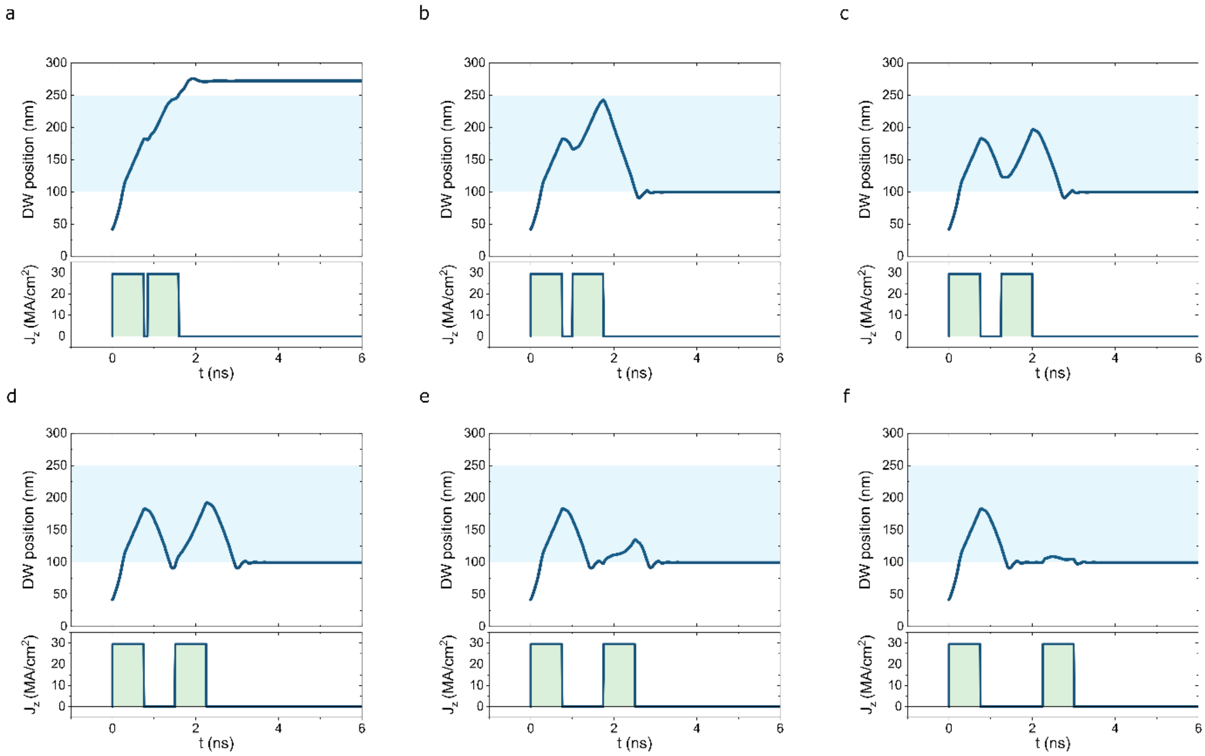

Figure S13. Pause time dependent leaky-integrate and fire behavior with two consecutive pulses. The pause time (time between the 2 current pulses) is adjusted from (a), 0.1 ns to (f), 1.5 ns with fixed pulse width of 0.75 ns.

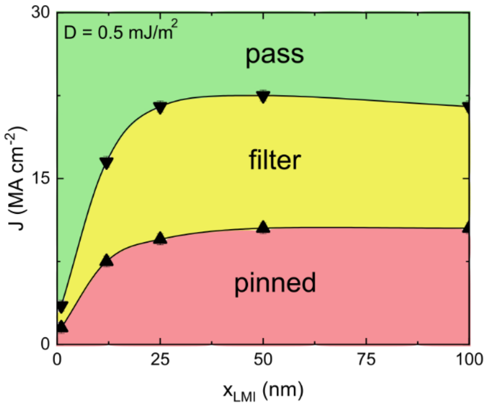

Figure S14. Micromagnetic simulation of DW filter operation for D = 0.5 mJ/m².

Supplementary Movie 1.

The results of a drift velocity experiment in micromagnetic simulations are presented here for illustrative purposes. The left column depicts the magnetic configuration of the inhibitor layer and the DW in the racetrack in side view. The simulated magnetization of the system in top view is shown in the right column. The bright (dark) contrast symbolises magnetisation ↑ (↓). The four rows illustrate the possible configurations: ↑↓ DW with inhibitor magnetized ↑, ↑↓ DW with inhibitor magnetized ↓, ↓↑ DW with inhibitor magnetized ↑, and ↓↑ DW with inhibitor magnetized ↓. The green (orange) arrow represents the current pulse applied along the device length x, in positive (negative) direction.

Supplementary Movie 2.

Micromagnetic simulations of the LIF functionality are presented here. The bottom right schematic defines the pause time, τ_{pause} and pulse time, τ_{pulse}. The simulations for τ_{pause} = 0.1 ns and four different values of τ_{pause} between 0.7 and 1.4 ns are shown. The top right graph shows the DW position as a function of time, for the four different τ_{pulse} considered. The light blue shaded area represents the region underneath the inhibitor. The magnetization of the system in top view is shown as a function of time in the central column. One frame corresponds to 0.1 ns. Parameters used in simulation are given in the left column. It should be noted that in the present case, for τ_{pause} = 0.1 ns, the observed non-linear behavior is a consequence of the combined effects of domain wall tilting and domain wall inertia. This can be clearly observed when comparing τ_{pulse} = 0.8 ns, for which we obtain LIF behavior, and τ_{pulse} = 1.0 ns, for which the DW cannot pass the inhibitor despite the longer pulse length. We attribute this phenomenon to the intricate interaction between stray field, domain wall inertia, and DW acceleration.
